# Supplementary material for: Highly sensitive visual restoration and protection via ectopic expression of chimeric rhodopsin in mice
Source: iScience. 2023 Aug 25;26(10):107716. doi: 10.1016/j.isci.2023.107716 (PMC10504486; doi:10.1016/j.isci.2023.107716)
Supplement: Document S1. Figures S1–S4 [file mmc1.pdf]

iScience, Volume *26*

Supplemental information

Highly sensitive visual restoration and protection via ectopic expression of chimeric rhodopsin in mice

Yusaku Katada, Kazuho Yoshida, Naho Serizawa, Deokho Lee, Kenta Kobayashi, Kazuno Negishi, Hideyuki Okano, Hideki Kandori, Kazuo Tsubota, and Toshihide Kurihara

Supplemental Figures

a

```
                                                       TM1                       TM2
GR       MLMTVFSSAPELALLGSTFAQVDPSNLSVSDSLTYGQFNLVYNAFSFAIAAMFASALFFFSAQALVGQRYRLALLVSAIV  80
GHCR     MLMTVFSSAPELALLGSTFAQVDPSNLSVSDSLTYGQFNLVYNAFSFAIAAMFASALFFFSAQALVGQRYRLALLVSAIV  80
coGHCR   MLMTVFSSAPELALLGSTFAQVDPSNLSVSDSLTYGQFNLVYNAFSFAIAAMFASALFFFSAQALVGQRYRLALLVSAIV  80

           TM2                                TM3                                  TM4
GR       VSIAGYHYFRIFNSWDAAYVLENGVYSLTSEKFNDAYRYVDWLLTVPLLLVETVAVL-----------TLPAKEARPLLI  149
GHCR     VSIAGYHYFRIFNSWDAAYVLENGVYSLTSEKFNDAYRYVDWLLTVPLLLVQTVAVIERYVVVCKPMSNFRFGENHPLLI  160
coGHCR   VSIAGYHYFRIFNSWDAAYVLENGVYSLTSEKFNDAYRYVDWLLTVPLLLVQTVAVIERYVVVCKPMSNFRFGENHPLLI  160
                                                                   Human rhodopsin

           TM4                      TM5                                        TM6
GR       KLTVASVLMIATGYPGEISDDITTRIIWGTVSTIPFAYILYVLWVELSRSLVRQPAA---------VQTLVRNMRWLLLL  220
GHCR     KLTVASVLMIATGYPGEISDDITTRIIWGTVSTIPFAYILYVLWVELSFT-VKEAAAQQQESATTQVQTLVRNMRWLLLL  239
coGHCR   KLTVASVLMIATGYPGEISDDITTRIIWGTVSTIPFAYILYVLWVELSFT-VKEAAAQQQESATTQVQTLVRNMRWLLLL  239
                                                         Human rhodopsin

          TM6                  TM7
GR       SWGVYPIAYLLPMLGVSGTSAAVGVQVGYTIADVLAKPVFGLLVFAIALVKTKADQESSEPHAAIGAAANKSGGSLIS--  298
GHCR     SWGVYPIAYLLPMLGVSGTSAAVGVQVGYTIADVLAKPVFGLLVFAIALVKTKADQESSEPHAAIGAAANKSGGSLIS--  317
coGHCR   SWGVYPIAYLLPMLGVSGTSAAVGVQVGYTIADVLAKPVFGLLVFAIALVKTKADQESSEPHAAIGAAANKSGGSLISGS  319


GR       -------------------------
GHCR     -------------------------
coGHCR   ASASNGASDYKDDDDKEFFCYENEV   344
                 FLAG      ER2
```

b

GHCR

AUGUUGAUGACCGUAUUUUCUUCUGCACCUGAACUUGCCCUUCUCGGAUCAACCUUUGCCCAGGUCGAUCCUUCAAACUUAUCGGUCU
CAGAUUCGCUGACCUAUGGUCAGUUCAAUCUGGUUUACAACGCUUUCUCGUUUGCCAUCGCGGCAAUGUUCGCAUCUGCCCUCUUCU
UCUUCAGCGCUCAGGCACUCGUCGGUCAACGAUACCGGUUGGCCUUGCUUGUUUCAGCAAUUGUUGUGAGUAUCGCUGGGUACCACU
ACUUUCGGAUCUUCAAUAGUUGGGAUGCUGCCUACGUUCUGGAGAAUGGCGUGUAUUCCCUGACUAGCGAAAAAUUCAACGACGCCUA
CCGCUAUGUGGAUUGGCUGUUGACCGUGCCUCUGUUGCUGGUGCAAACAGUGGCAGUGAUCGAGCGGUACGUAGUGGUGUGCAAGC
CCAUGAGCAACUUCCGCUUCGGGGAGAACCACCCCUUGCUGAUCAAACUGACGGUGGCUUCAGUUCUGAUGAUUGCCACGGGCUACC
CCGGCGAGAUUUCUGACGACAUUACGACUCGCAUCAUCUGGGGUACGGUCAGCACGAUUCCCUUCGCCUACAUCCUCUAUGUGUUGU
GGGUCGAACUGUCCUUCACCGUCAAGGAGGCUGCAGCCCAGCAGCAGGAGUCGGCCACCACUCAGGUACAAACCCUGGUCCGCAACA
UGCGGUGGCUGCUGUUGCUCUCCUGGGGUGUUUACCCGAUCGCAUACCUUCUACCCAUGCUUGGAGUAUCCGGUACGUCCGCGGCU
GUCGGCGUUCAGGUUGGCUAUACGAUCGCAGACGUGCUGGCGAAGCCUGUAUUUGGUCUUCUAGUCUUCGCGAUUGCACUCGUGAAA
ACAAAAGCAGAUCAAGAAAGCAGUGAACCACAUGCCGCAAUAGGUGCUGCUGCAAAUAAAUCGGGAGGCAGUCUUAUCUCCUGA

coGHCR

AUGCUGAUGACCGUGUUCAGCAGCGCCCCUGAGCUGGCCCUGCUGGGCUCUACAUUUGCCCAGGUGGACCCCAGCAACCUGAGCGUG
UCCGACAGCCUGACCUACGGCCAGUUCAACCUGGUGUACAACGCCUUCAGCUUCGCCAUUGCCGCCAUGUUCGCCAGCGCCCUGUUC
UUCUUCAGUGCACAAGCCCUCGUGGGCCAGCGGUACAGACUGGCUCUGCUGGUGUCUGCCAUCGUGGUGUCUAUCGCCGGCUACCA
CUACUUCCGGAUCUUCAACAGCUGGGACGCCGCCUACGUGCUGGAAAACGGCGUGUACUCCCUGACCAGCGAGAAGUUCAACGACGC
CUACAGAUACGUGGACUGGCUGCUGACCGUGCCCCUGCUGCUGGUGCAAACAGUGGCCGUGAUCGAGCGCUACGUGGUCGUGUGCA
AGCCCAUGAGCAACUUCAGAUUCGGCGAGAACCACCCCCUGCUGAUCAAGCUGACAGUGGCCAGCGUGCUGAUGAUCGCCACAGGCU
ACCCUGGCGAGAUCAGCGACGACAUCACCACCCGGAUCAUCUGGGGAACCGUGUCCACCAUCCCCUUCGCCUACAUCCUGUACGUGCU
GUGGGUGGAACUGAGCUUCACCGUGAAAGAGGCCGCUGCUCAGCAGCAGGAAAGCGCCACAACCCAGGUGCAGACCCUCGUGCGGAA
CAUGAGAUGGUUGCUGCUGCUGUCCUGGGGCGUGUACCCUAUCGCCUACCUGCUGCCUAUGCUGGGCGUGUCCGGAACAUCUGCCG
CUGUGGGAGUGCAAGUGGGCUACACAAUCGCCGAUGUGCUGGCCAAGCCCGUGUUCGGCCUGCUGGUGUUCGCUAUCGCCCUCGUG
AAAACAAAGGCCGACCAGGAAAGCAGCGAGCCCCACGCCGCUAUUGGAGCCGCCGCUAACAAGUCUGGCGGCAGCCUGAUCAGCGGA
UCCGCCAGCGCCAGCAACGGCGCCAGCGACUACAAAGACGAUGACGACAAGGAAUUCUUCUGCUACGAGAAUGAAGUGUAA

Figure S1. GHCR structural alignment, related to Figure 1.

(a) Primary structure alignment of wild type GR, GHCR, and coGHCR. GR, *Gloeobacter* rhodopsin; GHCR, *Gloeobacter* and human chimeric rhodopsin; coGHCR, codon-optimized *Gloeobacter* and human chimeric rhodopsin. Gray, E132Q mutation; green, inserted constructs of the second and third loops of human rhodopsin. TM, transmembrane domains; FLAG, FLAG tag; ER2, ER2 ER export signal. (b) RNA sequences of GHCR and coGHCR.

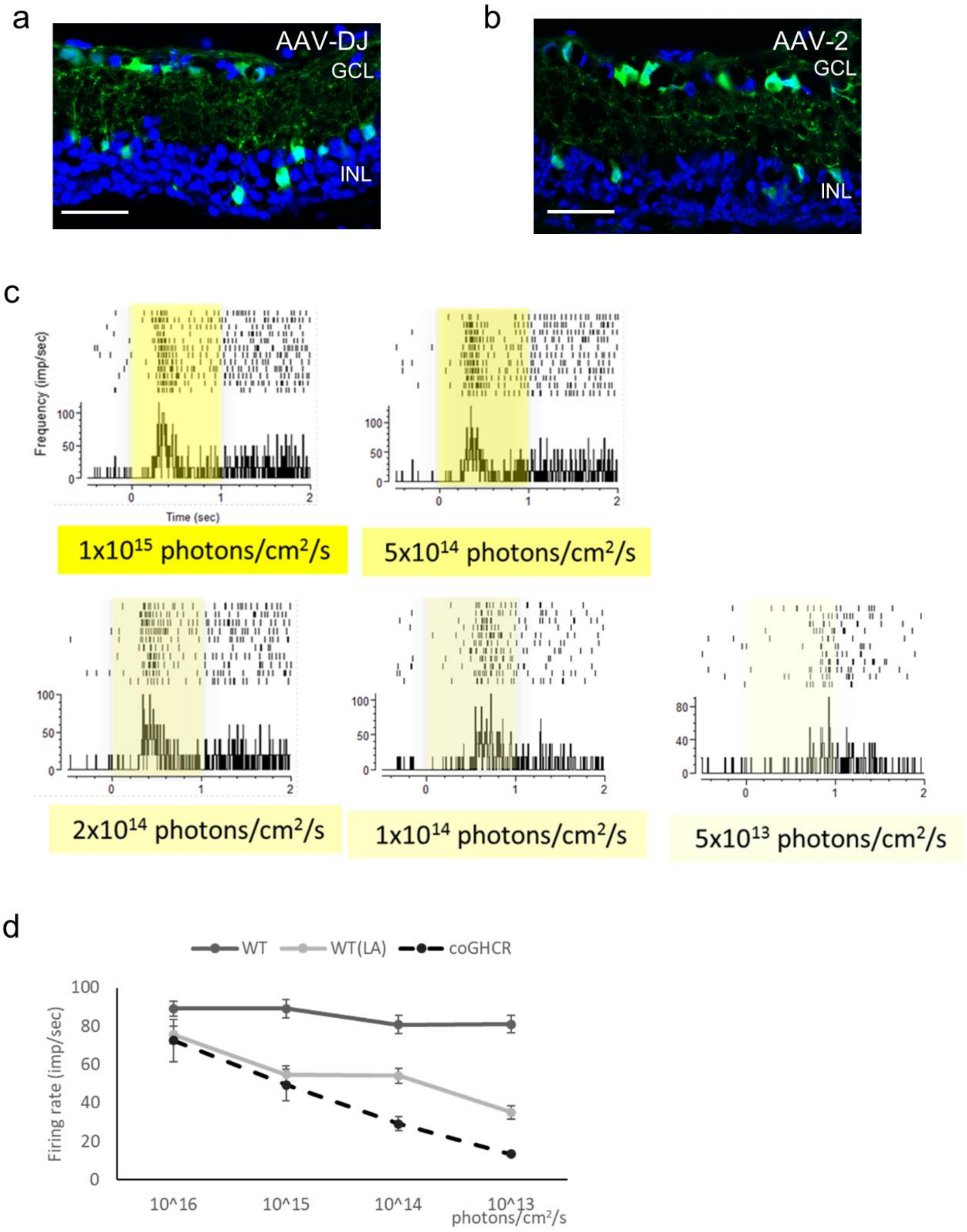
a
AAV-DJ
GCL
INL
b
AAV-2
GCL
INL
c
Frequency (imp/sec)
Time (sec)
1x10^15 photons/cm²/s
5x10^14 photons/cm²/s
2x10^14 photons/cm²/s
1x10^14 photons/cm²/s
5x10^13 photons/cm²/s
d
WT
WT(LA)
coGHCR
Firing rate (imp/sec)
10^16
10^15
10^14
10^13
photons/cm²/s

Figure S2. Ectopic expression of AAV-2-CAGGS-GHCR restores light responses in the *rd1* mouse retina, related to Figure 1.

(a, b) Confocal images of transverse sections of a C57BL/6 retina 2 months after AAV-DJ-CAGGS-EGFP (a) and AAV-2-CAGGS-EGFP (b) intravitreal injection. Blue, DAPI nuclear counterstaining. Scale bar, 50 μm. (c) Raster plots and peri-stimulus time histogram of light stimulation of GHCR-treated (AAV-2-CAGGS-GHCR) mice. The response to stimulation with a white LED with varying light intensity (irradiation duration, 1.0 s) was recorded. (d) Quantification of firing rates from RGCs in wild-type mice, light-adapted (LA) wild-type mice and rd1 mouse retina 2 after AAV-DJ-CAGGS-coGHCR intravitreal injection at the indicated light intensity. GCL, ganglion cell layer; INL, inner nuclear layer. coGHCR, codon-optimized *Gloeobacter* and human chimeric rhodopsin.

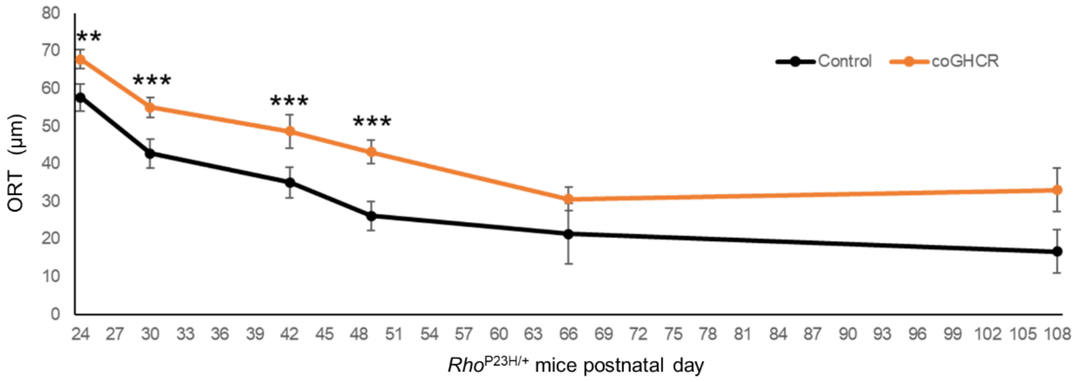

Figure S3. Characterization of coGHCR-mediated retinal protection via OCT imaging, related to Figure 4.

Follow-up of ORT (from ONL to cone outer segment) after AAV-DJ-CAGGS-coGHCR and control (AAV-DJ-CAGGS-EGFP) subretinal injection into postnatal day (PND) 0–1 *Rho*$^{P23H/+}$ mouse retinas, measured with OCT. Error bars represent SEM. Data were analyzed with the unpaired t-test; ** represents $p < 0.01$ and *** represents $p < 0.001$. coGHCR, codon-optimized *Gloeobacter* and human chimeric rhodopsin; ORT, outer retinal thickness.

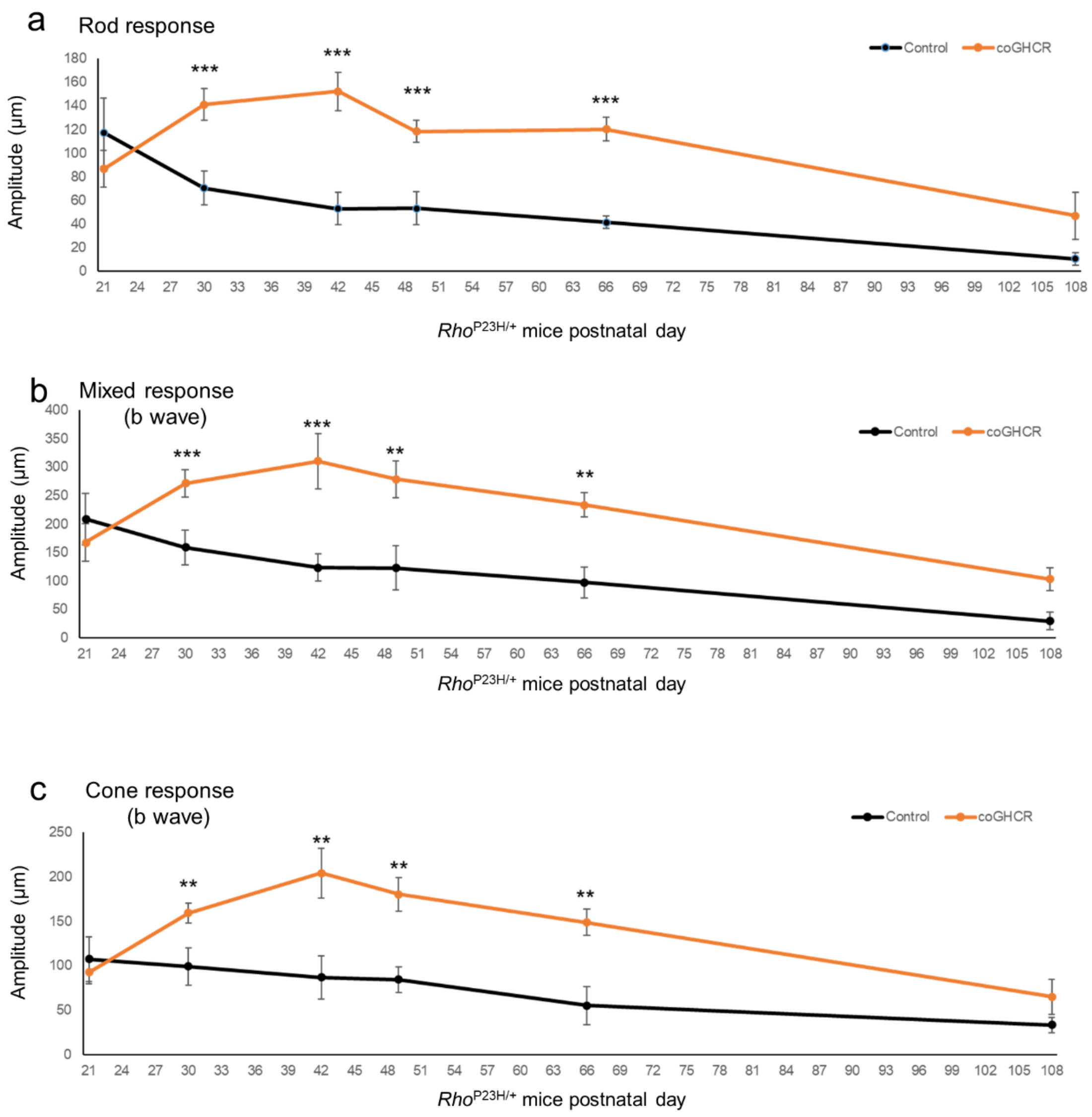

Figure S4. Characterization of coGHCR-mediated retinal protection as assessed by ERG recording, related to Figure 4.

(a–c) Follow-up of the ERG amplitude of the rod response (a), the mixed b wave (b), and the cone b wave (c) after AAV-DJ-CAGGS-coGHCR and control (AAV-DJ-CAGGS-EGFP) subretinal injection into postnatal day (PND) 0–1 *Rho*$^{P23H/+}$ mouse retinas. Error bars represent SEM. Data were analyzed with the unpaired t-test; ** represents $p < 0.01$ and *** represents $p < 0.001$. coGHCR, codon-optimized *Gloeobacter* and human chimeric rhodopsin.
